# Calpain-2 Inhibitors as Therapy for Traumatic Brain Injury

**DOI:** 10.1007/s13311-023-01407-y

**Published:** 2023-07-20

**Authors:** Michel Baudry, Yun Lyna Luo, Xiaoning Bi

**Affiliations:** 1 CDM, Western University of Health Sciences, 309 E. 2nd St, Pomona, CA 91766 USA; 2 CoP, Western University of Health Sciences, Pomona, CA 91766 USA; 3 COMP, Western University of Health Sciences, Pomona, CA 91766 USA

**Keywords:** Calpain, Concussion, Neurodegeneration, Biomarker, Learning and memory

## Abstract

While calpains have long been implicated in neurodegeneration, no calpain inhibitor has been developed for the treatment of neurodegeneration. This is partly due to the lack of understanding of the specific functions of most of the 15 members of the calpain family. Work from our laboratory over the last 5–10 years has revealed that calpain-1 and calpain-2, two of the major calpain isoforms in the brain, play opposite roles in both synaptic plasticity/learning and memory and neuroprotection/neurodegeneration. Thus, calpain-1 activation is required for triggering certain forms of synaptic plasticity and for learning some types of information and is neuroprotective. In contrast, calpain-2 activation limits the extent of synaptic plasticity and of learning and is neurodegenerative. These results have been validated with the use of calpain-1 knock-out mice and mice with a selective calpain-2 deletion in excitatory neurons of the forebrain. Through a medicinal chemistry campaign, we have identified a number of selective calpain-2 inhibitors and shown that these inhibitors do facilitate learning of certain tasks and are neuroprotective in a number of animal models of acute neurodegeneration. One of these inhibitors, NA-184, is currently being developed for the treatment of traumatic brain injury, and clinical trials are being planned.

Traumatic brain injury (TBI) is a significant public health problem in the USA. It is estimated that 2.8 million TBI cases are reported every year in the USA and it is likely that many more cases are never reported (https://www.cdc.gov/traumaticbraininjury/get_the_facts.html). The cause of injury varies greatly and includes motor vehicle accidents, falls, sport injuries, and gunshot wounds, to name a few. The severity of TBI is generally classified as mild (mTBI), also called concussion, moderate, and severe, which is often associated with a prolonged period of unconsciousness after the injury.

It is now well established that glutamate excitotoxicity is a critical mechanism triggering neurodegeneration in the CNS [1]. Excitotoxicity is caused by excessive and prolonged glutamate release resulting from disruption of the homeostatic mechanisms that control glutamate release and reuptake. Several mechanisms that play important roles in neuronal death as a result of excitotoxicity have been identified and could lead to the development of new strategies for neuroprotection [1]. Particular emphasis has been placed on the roles of a subtype of glutamate receptors, the NMDA receptors, as their activation results in calcium influx and initiation of a variety of calcium-dependent cascades. While initial studies focused on developing NMDA receptor antagonists as potential neuroprotective compounds, the multitude of failures of such compounds in clinical trials has shifted the emphasis to targets downstream of these receptors [2]. Moreover, synaptic and extrasynaptic NMDA receptors play opposite functions in glutamate excitotoxicity [3, 4]. Thus, activation of synaptic NMDA receptors produces neuroprotection, while activation of extrasynaptic NMDA receptors leads to neurodegeneration. There is, therefore, a strong interest in identifying key targets downstream of extrasynaptic NMDA receptors.

Calpain has long been shown to be involved in neurodegeneration [5, 6] in general and in stroke [7, 8] and TBI [9, 10] in particular. Consequently, numerous studies have attempted to use calpain inhibitors to reduce neurodegeneration in both stroke and TBI [10–12]. While some studies have reported positive effects of the first generation of calpain inhibitors in TBI [13], other studies have not [14, 15]. In particular, administration of the ketoamide calpain inhibitor, AK295, 15 min after a lateral fluid percussion brain injury reduced the cognitive and motor dysfunction [16], but had no effect on neuronal damage and on calpain activation assessed by measuring the levels of spectrin degradation [17]. Preinjury administration of the non-selective calpain inhibitor, MDL-28710, also reduced the short-term diffuse axonal injury in a rat model of impact acceleration head injury [18]. The same calpain inhibitor was also found to be neuroprotective for the corpus callosum in a rat model of fluid percussion injury. The time window for neuroprotection was 4 h and repeated injections provided long-term protection [19]. On the other hand, MDL-28710 did not prevent alterations in axonal transport following axonal stretch injury [20]. Recent studies concluded that even two blood–brain barrier and cell-permeable calpain inhibitors, SNJ-1945 and MDL-28170, did not have sufficient efficacy or a practical therapeutic window in a model of controlled cortical impact (CCI) [13, 14]. While those non-isoform selective calpain inhibitors were shown to inhibit overall calpain activation (without distinguishing which calpain isoform was targeted) following TBI, they failed to provide neuroprotection. Several reasons could account for the failure to develop clinical applications with such inhibitors, including their lack of specificity/potency/selectivity [21] and the incomplete knowledge regarding the functions of the major calpain isoforms in the brain, e.g., calpain-1 and calpain-2 (aka µ- and m-calpain). Work from our laboratory over the last 5–10 years has revealed that calpain-1 and calpain-2 play opposite roles in both synaptic plasticity and neuroprotection/neurodegeneration [22]. Thus, calpain-1 activation is required for theta burst stimulation-induced long-term potentiation (LTP) and is neuroprotective [23, 24]. On the other hand, calpain-2 activation limits the magnitude of LTP and is neurodegenerative [23, 24]. These findings could explain the failure of the previous studies to convincingly demonstrate the role of calpain in neurodegeneration and the lack of clear efficacy of the previously tested calpain inhibitors, which did not discriminate between calpain-1 and calpain-2. Based on the many studies we and others have published over the last 5–10 years, we have proposed a model accounting for the differential activation of calpain-1 and calpain-2 in the brain following various types of acute injury, including TBI (Fig. 1). In our model, calpain-1 is rapidly and transiently activated, possibly downstream of synaptic NMDA receptors. Its activation leads to Akt activation and the transient stimulation of neuroprotective signaling mechanisms. This initial event is followed by the prolonged activation for several days of calpain-2 and the stimulation of neurodegenerative signaling pathways, including STEP and p38 [22]. Note that in both cases, the cytoskeletal protein, spectrin, is cleaved, making the calpain-mediated spectrin breakdown product (SBDP) a reliable marker for calpain activation. This review will summarize the evidence we have accumulated over the last 5–10 years supporting the use of selective calpain-2 inhibitors for the treatment of TBI/concussion.

**Fig. 1:**
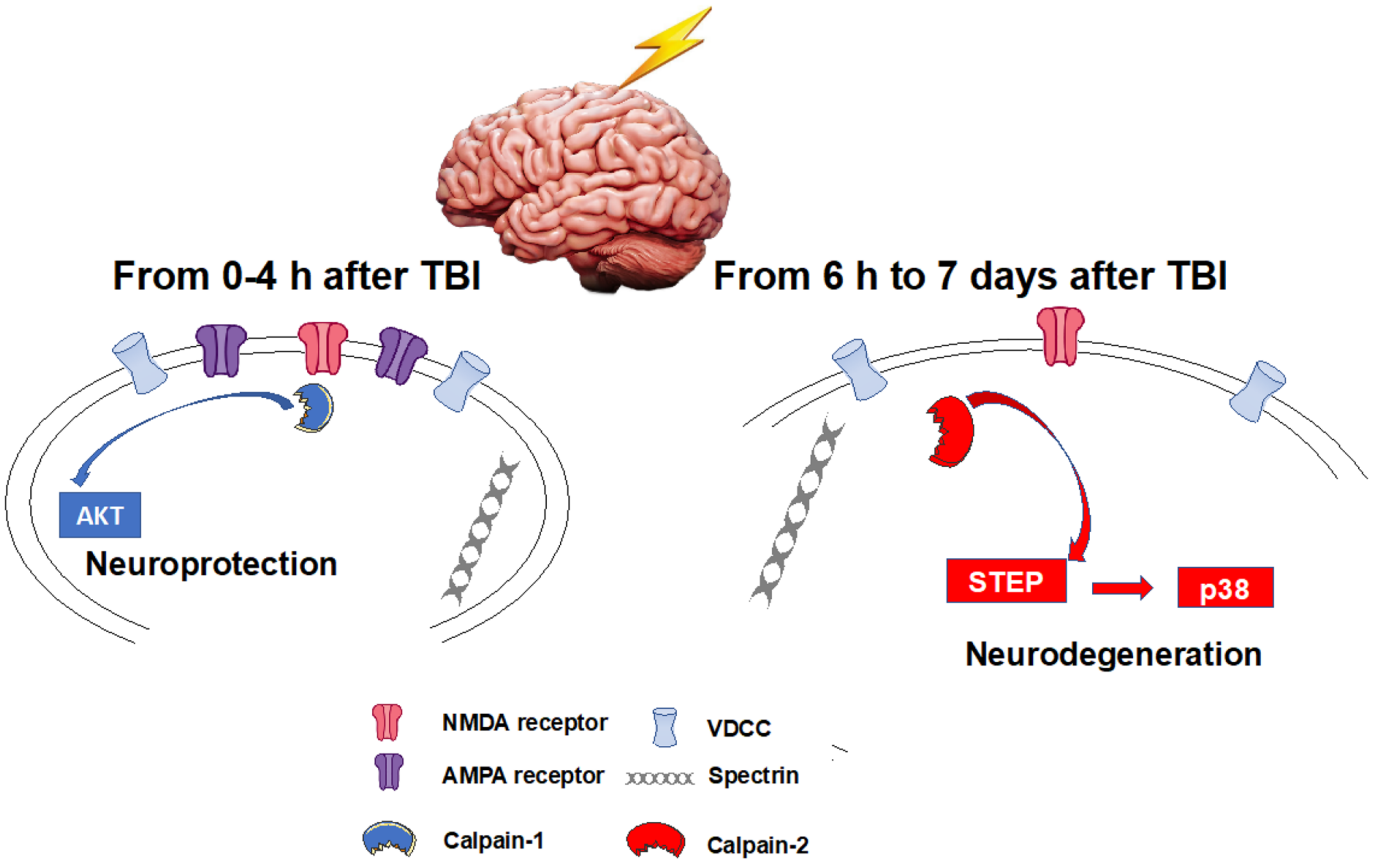
Schematic diagram summarizing the opposite functions of calpain-1 and calpain-2 following TBI. Calpain-1 is rapidly but transiently activated following TBI. Its activation results in the stimulation of neuroprotective pathways, including the Akt pathway. On the other hand, calpain-2 activation is delayed and prolonged lasting for several days after TBI. Its activation leads to the stimulation of neurodegenerative pathways, including STEP and p38. In both cases, spectrin is cleaved, generating a specific breakdown product (SBDP), which is widely used as an index of brain calpain activation. We are also postulating that calpain-1 is located postsynaptically downstream of the NMDA receptors, while calpain-2 is mostly located extrasynaptically. This differential localization of calpain-1 and calpain-2 is most likely due to the existence of different PDZ binding domains in calpain-1 and calpain-2 [22]

## Role of Calpain-2 in Acute TBI

Once we identified the opposite roles of calpain-1 and calpain-2 in neurodegeneration, we searched the literature for calpain inhibitors exhibiting more selectivity for calpain-2 than for calpain-1. We found one such compound in Li et al. [25], which consisted of the peptidyl α-ketoamide referred to as C18 (Fig. 1, Z-Leu-Abu-CONH-CH_2_-C_6_H_3_(3,5-(OMe)_2_)), with a reported 100-fold difference in Ki values for calpain-2 and calpain-1. We obtained this compound and found that under our conditions, the selectivity for calpain-2 or calpain-1, as assessed with the value of the ratio of the Ki for both enzymes, was highly dependent on the species used in the calpain assays (human, porcine, mice, rat). We renamed this compound C2I (calpain-2 inhibitor) or NA-101. We also identified the *S*-S-diastereomer at P1 as the active isomer while the R-S-diastereomer was inactive.

We used the controlled cortical impact (CCI) mouse model of TBI to test the effects of C2I on neurodegeneration in this model. These data have been published [26] and will be briefly summarized here. In order to determine the levels of in situ activation of the two major calpain isoforms in the brain following CCI, we performed immunohistochemistry (IHC) to label spectrin breakdown product (SBDP), a calpain activity marker generated by calpain-1 and calpain-2 truncation of αII-spectrin [27] in brain sections of WT and calpain-1 KO mice collected at multiple time points after CCI. SBDP levels in WT mice reflect the combined activity of both calpain-1 and calpain-2, while in calpain-1 KO mice they reflect only calpain-2 activation. SBDP levels were significantly increased at 2, 6, 8, 24, and 72 h after CCI around the lesion site in WT mice, while in calpain-1 KO mice they were not significantly elevated until 8 h after CCI. No significant difference in SBDP levels were observed between WT and calpain-1 KO mice at 24 and 72 h after CCI. These results indicated that calpain-1 is rapidly but transiently activated after the trauma, while calpain-2 activation is delayed and prolonged following TBI. These results were validated by using IHC with an antibody against phosphatase and tensin homolog (PTEN) in the same set of brain sections, as we previously showed that PTEN is a selective substrate for calpain-2 [28, 29]. Changes in PTEN-immunoreactivity (ir) exhibited a similar time course in WT and KO mice. In both genotypes, PTEN-ir was significant decreased at 8 h, exhibited a maximum decrease at 24 h after CCI, and slowly recovered but was still significantly decreased at 72 h. The decrease in PTEN-ir in WT and calpain-1 KO mice was inversely correlated with the increase in SBDP levels in calpain-1 KO mice. These results confirmed that calpain-2 is the calpain isoform that exhibits a prolonged activation following TBI, with no or little contribution of calpain-1 after a few hours after TBI.

**Fig. 2:**
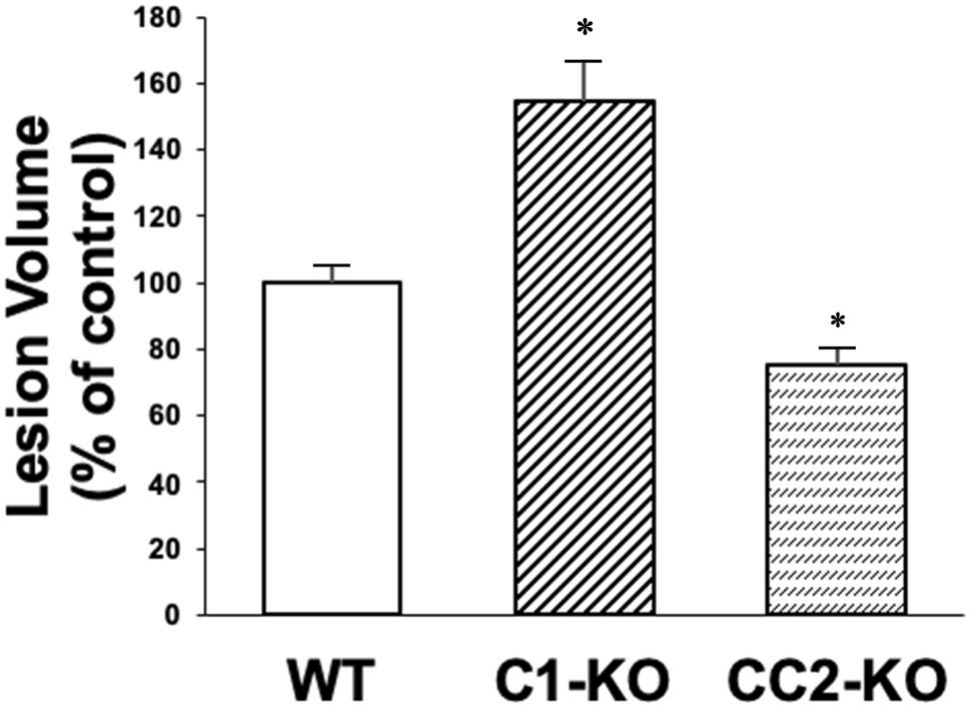
Lesion volume following TBI in wild-type, calpain-1 KO, and conditional calpain-2 KO mice. Wild-type (WT), calpain-1 knock-out (C1-KO), and conditional calpain-2 knock-out (CC2-KO) mice were subjected to the CCI model of TBI in separate experiments. Lesion volume was measured 3 days after TBI. Lesion area was measured in 8 brain sections from each animal. Total lesion volume was then measured by integrating the areas across all sections. Results are expressed as percent of the lesion volume in WT mice in the respective experiments and are means ± SEM of 8, 4, and 6 animals, respectively. *p < 0.05, as compared to WT mice. Data for the C1-KO mice are from [26] and for CC2-KO from [42]

We also compared the time course of cell death following TBI in WT and calpain-1 KO mice, using TUNEL staining in brain sections from WT and calpain-1 KO mice at various time points after CCI. The number of TUNEL-positive cells around the impact site was not significantly increased until 24 h after CCI in WT mice, while it was significantly increased as early as 6 h after CCI in calpain-1 KO mice. Moreover, the lesion volume measured 3 days after CCI was significantly larger in calpain-1 KO mice, as compared to WT mice (Fig. 2). In contrast, lesion volume in conditional calpain-2 knock-out mice, with calpain-2 deletion in excitatory neurons of the forebrain, was significantly reduced (Fig. 2). These results indicate that the lack of calpain-1 activity exacerbates neuronal damage following TBI, as we previously showed in a mouse model of acute glaucoma [29]. They also strongly support the idea that a non-selective calpain inhibitor may not be appropriate for treating various neurodegenerative diseases.

We analyzed the correlation between the levels of SBDP or PTEN around the lesion site in individual animal and the numbers of TUNEL-positive cells around the lesion site in the same animal. Interestingly, cell death was highly correlated with SBDP levels in calpain-1 KO mice, which reflect calpain-2 activation. In contrast, the correlation was not as clear in WT mice, which was expected since SBDP levels reflect both calpain-1 and calpain-2 activation. In addition, cell death was also highly correlated with PTEN levels, which reflect calpain-2 activity, in both WT and calpain-1 KO mice. These results strongly support the idea that the level of calpain-2 activation determines the extent of cell death around the lesion site after TBI.

We used the relatively selective calpain-2 inhibitor, NA-101, to further validate the role of calpain-2 in neuronal damage following TBI. Systemic administration of NA-101 significantly decreased SBDP levels and increased PTEN levels around the lesion site. It also significantly reduced the number of TUNEL-positive as well as the number of Fluoro-Jade C-positive cells around the lesion area [26]. In order to evaluate the window during which calpain-2 inhibition would be effective against the long-term consequences of TBI, NA-101 was administered only once 1 h after TBI or every day for 1 week after TBI. While a single injection of NA-101 1 h after TBI failed to provide long-term protective effect, repeated daily injection significantly reduced lesion volume 1 month after TBI, as compared to vehicle injections [26]. Interestingly, the lesion volume significantly increased between 3 days and 1 month after TBI, indicating that processes initiated by TBI continue to contribute to cell death for many days after TBI and that repetitive NA-101 treatment was effective at blocking this cascade. These results also suggest that the time window to inhibit calpain-2 following TBI is quite large, due to the prolonged activation of calpain-2.

We also determined the effects of NA-101 injection on the motor and cognitive impairments following TBI. Motor deficit and recovery after TBI were analyzed with the beam walking test, using the latency to cross and the number of foot slips of the left hindlimb (contralateral to the injury), as mice walked across a 1-m-long round beam. While there was no significant difference in time to cross or number of foot-slips between NA-101- and vehicle-treated groups on day 1, animals treated with NA-101 (0.3 mg/kg 1 h after TBI) rapidly recovered motor functions to levels similar to those in naive and sham mice, while vehicle-treated mice recovered much slower. Furthermore, motor function was significantly improved 8 days after TBI in mice injected daily with NA-101 during day 0–7 after TBI, as compared to vehicle-treated mice [26]. We used the fear conditioning paradigm to analyze the effect of repeated daily injection of NA-101 for 7 days on TBI-induced learning impairment. Context-dependent but not tone-dependent learning was impaired after TBI in vehicle-treated mice, and NA-101 treatment after TBI rescued context learning. These results indicate that daily injection of NA-101 for 7 days after TBI reverses TBI-induced impairment in both motor and cognitive functions. We also showed that NA-101 was effective to inhibit calpain-2 activation with intravenous (i.v.) injection [26].

Blood levels of a calpain-mediated fragment of brain spectrin, spectrin N-terminal fragment (SNTF) (spectrin N-terminal fragment), represent a potential diagnostic tool for TBI [30, 31]. In human TBI, serum SNTF levels are significantly elevated 24 h after concussion and the values are predictive of the long-term cognitive and motor sensory dysfunction analyzed at 90 days after concussion [32]. In the CCI mouse model of TBI, blood levels of SNTF were increased 24 h after the trauma and this increase was prevented by treatment with NA-101 [33]. Our laboratory has also identified another blood biomarker, which reflects brain activation of calpain-2. It represents a calpain-2-mediated fragment of the tyrosine phosphatase, PTPN13, which we referred to as P13BP [34]. P13BP levels are also elevated in blood 24 h after TBI in both the mouse model of TBI and in human subjects. Moreover, blood P13BP levels remain elevated for several days after TBI in the mouse model and correlate with brain calpain-2 activation [34]. In human TBI patients, blood levels of P13BP also correlate with the severity of the trauma assessed with the Glasgow Coma Scale (GCS) score [34].

To summarize, our results have clearly demonstrated that calpain-2 activation remains elevated for days after TBI and is responsible for the brain damage and associated motor and cognitive impairments resulting from the trauma. We further demonstrated that a relatively selective calpain-2 inhibitor prevented calpain-2 activation, reduced neuronal damage, prevented motor and cognitive impairment, and reduced the levels of blood biomarkers reflecting brain calpain-2 activation.

## Role of Calpain-2 in Repeated Mild TBI

In recent years, repeated mild traumatic brain injury (rmTBI) has received a lot of attention after it was found that a significant number of athletes subjected to repeated concussions exhibit a chronic degenerative disease referred to as chronic traumatic encephalopathy [35]. CTE is characterized by massive accumulation of hyperphosphorylated tau, gliosis, and neurodegeneration [36]. Diffuse axonal degeneration has been shown to be responsible for many of the long-term functional consequences of mTBI [37, 38], and calpain is widely thought to be involved in diffuse axonal injury. Furthermore, blood levels of calpain-mediated SNTF are elevated shortly after injury and predict the long-term consequences of the injury in patients with mTBI, including professional hockey players experiencing concussions [30, 39]. While there is good evidence for a role of calpain in mTBI, which calpain isoform is responsible for producing the neuropathological consequences of mTBI or rmTBI had not been established. We used the repetitive concussion model developed by Petraglia and colleagues [40, 41]; in this model, awake mice are subjected to 4 daily hits on the head for 10 consecutive days. Previous studies using this model of repeated concussions have shown that mice exhibited a number of behavioral impairments, including cognitive impairment, as well as many pathological changes, such as astrocyte and microglia activation in various brain regions, as well as axonal degeneration, mainly in the corpus callosum and the optic tract [40]. To analyze the contribution of calpain-2 in mTBI and rmTBI, we developed a calpain-2 conditional knock-out (C2CKO) mouse by crossing calpain-2 floxed mice with Cre-CamKII mice, which produced mice with calpain-2 deletion in all excitatory neurons of the forebrain, and we also tested the effects of NA-101 in this model. Since repeated concussions were administered over a period of 10 days, we chose to deliver NA-101 through subcutaneously implanted Alzet mini-pumps. The pumps were implanted the day before the start of the concussions and were taken out after 2 weeks. These results have been recently published [42] and will only be briefly summarized here.

SBDP levels in the cortex ipsilateral to the impact side were elevated at 24 h and 3 days after the last impact and remained slightly elevated at 7 days after the last impact. In contrast, there was no increase in SBDP levels at any time in cortex or hippocampus from C2CKO mice or following daily injections of NA-101 for 10 days. We also determined changes in levels of the phospho-phosphatase-activated domain (p-PAD) of tau, which is present early in tauopathy [43, 44]. In WT mice, the changes in p-PAD-tau were quite similar to those found for SBDP in both cortex and hippocampus. However, there were no changes in p-PAD-tau in cortex and hippocampus from C2CKO mice after rmTBI or following NA-101 injections.

At 1 and 3 months after the last concussion, WT mice exhibited depressed behavior, as evidenced in the tail suspension test. They also exhibited increased risk-taking behavior in the elevated plus maze [42]. In contrast, C2CKO mice or mice injected with NA-101 did not exhibit any of these behavioral alterations. Cognitive function was assessed at 1 and 3 months after repeated concussions using hippocampus-dependent fear conditioning. While WT mice exhibited significant impairment in learning and memory at both time points, C2CKO mice or mice injected with NA-101 did not exhibit any significant deficits.

A major pathological signature of repeated concussions is brain inflammation reflected by astrocyte and microglia activation [37]. Brain astrocyte and microglia activation was analyzed 3 months following repeated concussions by using immunohistochemistry to label GFAP-positive astrocytes and Iba-1-positive microglia, two widely used markers for activated astrocytes and microglia [45, 46]. While the numbers of reactive astrocytes and activated microglia were significantly increased in hippocampus and cortex of WT mice, C2CKO mice or mice injected with NA-1–1 did not exhibit any significant increase.

As mentioned above, another pathological feature of repeated concussions is axonal degeneration in the corpus callosum and optic tract [37]. Axonal degeneration was visualized by using the Gallyas staining, which revealed prominent degeneration in the optic tract in WT mice subjected to rmTBI, but no significant axonal degeneration was observed in C2CKO mice or mice injected with NA-101 subjected to rmTBI. Neuronal loss has also been observed in some models of repeated concussions [47].

Another hallmark of CTE is a massive increase in tau hyperphosphorylation at various residues in various brain regions. In our previous studies using the CCI mouse model of TBI, we also found massive increase in tau phosphorylation in cortex and showed that this effect was triggered at least in part by calpain-2-mediated cleavage of the tyrosine phosphatase, PTPN13, and the resulting activation of c-Abl [26]. In the rmTBI model, massive increase in tau phosphorylation at threonine 231 was also present in cortex, corpus callosum, and optic tract at 3 months after rmTBI in WT mice, but no significant changes in tau phosphorylation were detected in C2CKO mice or mice injected with NA-101 subjected to rmTBI. TDP-43 is an RNA/DNA binding protein, which accumulates in neurons in ALS and frontotemporal lobar degeneration [48]. It has been proposed that TDP-43 accumulation in these diseases is due to its partial cleavage by calpain, preventing its nuclear transport, resulting in its cytosolic accumulation and aggregation [49]. Cortical levels of TDP-43 following rmTBI were significantly decreased at 1, 3, and 7 days after repeated concussions in WT mice but were unchanged in C2CKO mice. Moreover, p-TDP-43 exhibited changes in subcellular localization, with accumulation in the cytoplasm and decreased levels in the nucleus, which were very similar to what has been reported in human patients with ALS or FTLD [48]. These changes in p-TDP-43 localization were completely absent in C2CKO mice and mice injected with NA-101.

All these results clearly establish that calpain-2 activation is a critical step leading to a wide range of neuropathological changes and behavioral alterations following either acute severe TBI or repeated mild TBI. They also demonstrate that a relatively short treatment with a selective calpain-2 inhibitor initiated shortly after concussion represents a novel therapeutic approach to prevent brain damage and behavioral modifications resulting from both severe and acute as well as mild repeated concussions. Interestingly, a couple of blood biomarkers reflect brain calpain activation, SNTF and a fragment of a selective target of calpain-2, the tyrosine phosphatase PTPN13, which we referred to as P13BP [34]. In addition, serum levels of Nfl have been shown to reflect axonal damage and to be elevated in a number of conditions associated with neuronal damage, including TBI [50]. All these blood biomarkers will be extremely useful to determine the potential efficacy of a selective calpain-2 inhibitor in human clinical trials.

## Developing New Analogs of NA-101

**Fig. 3:**
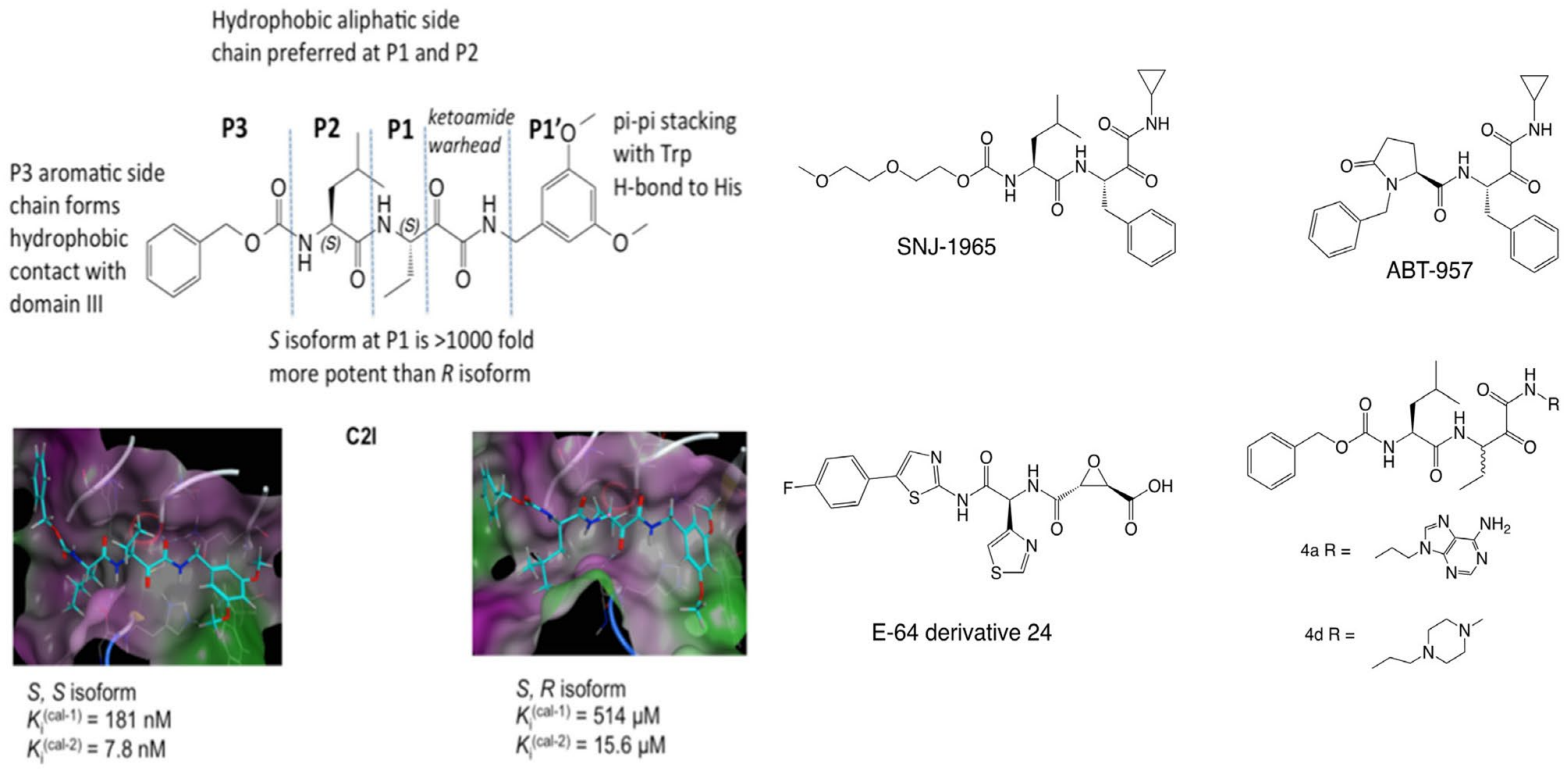
Comparison of C2I/NA-101 with other calpain inhibitors. C2I shares a similar scaffold with the other compounds shown in the figure. SNJ-1945, ABT-957, 4a, and 4d, all incorporate α-ketoamide warheads. SNJ-1945 has been reported as BBB permeable through LC–MS/MS [58]; ABT-957 was shown to cross rat BBB after intraperitoneal injection and was in clinical trial phase I for AD [59, 60]; and both 4a and 4d have therapeutically useful concentrations in mouse brain after subcutaneous administration [61]. An E-64 derivative has been reported to effectively penetrate the brain and significantly inhibit calpain-catalyzed hydrolysis of spectrin [62]. However, none of these calpain inhibitors shows calpain-2 selectivity, except C2I. Also shown is the location of the two chiral centers and the docking of the two diastereoisomers, S,S-C2I and S,R-C2I, in the catalytic site of calpain-2, with their respective Ki against calpain-1 (cal-1) or calpain-2 (cal-2)

While NA-101 showed interesting pharmacological properties, it was not suitable for clinical development for a variety of reasons. NA-101 scored −13.6 for drug-likeness using the OSIRIS Datawarrior software, and most developed drugs have positive drug-likeness scores. Moreover, NA-101 exhibited an inverted U-shape dose-response in the CCI model of TBI, as low doses provided neuroprotection while high doses exacerbated neuronal damage [26]. Therefore, we initiated a program to identify better analogs and ultimately a lead clinical candidate. The structure of NA-101 is shown in Fig. 3, which indicates that this compound shares a similar scaffold as other calpain inhibitors, some of which have been tested in clinical trials (ABT-957 by AbbVie) or are close to being tested in clinical trials (SNJ-1945 by Senju). Also, several α-ketoamide inhibitors of serine proteases or anti-inflammatory agents have been studied in clinical trials, and some are marketed (telaprevir, boceprevir, and tacrolimus). Many dipeptide analogs with MW values higher than 700 are known drugs or have been clinically tested (simeprevir, vaniprevir, and faldaprevir). NA-101 is a peptidyl α-ketoamide that binds to the catalytic triad of the calpain protease core as a covalent, reversible inhibitor. Among several reversible calpain inhibitors, α-ketoamides carry the most cytosolic stable electrophilic warhead [25, 51], and therefore are promising candidates for developing clinical therapeutics. Molecular dynamics simulations showed that NA-101 in calpain-1/2 adopts different binding poses when the less conserved domain III of calpain, in addition to the catalytic domain I and II, is included in the simulations. Compared with the binding pose in calpain-2, the binding pose in calpain-1 lacks the hydrogen bond between the catalytic histidine and a P1’ methoxy group.

This finding indicates that the isoform difference in domain III can propagate to the prime site. It was therefore beneficial to keep the NA-101 scaffold and focus on the lead-optimization at the P3 and P1’ positions to increase isoform selectivity. To predict the calpain isoform selectivity, we developed computational approaches to rank the reversible covalent binders, such as ketoamide compounds [52–54].

**Fig. 4:**
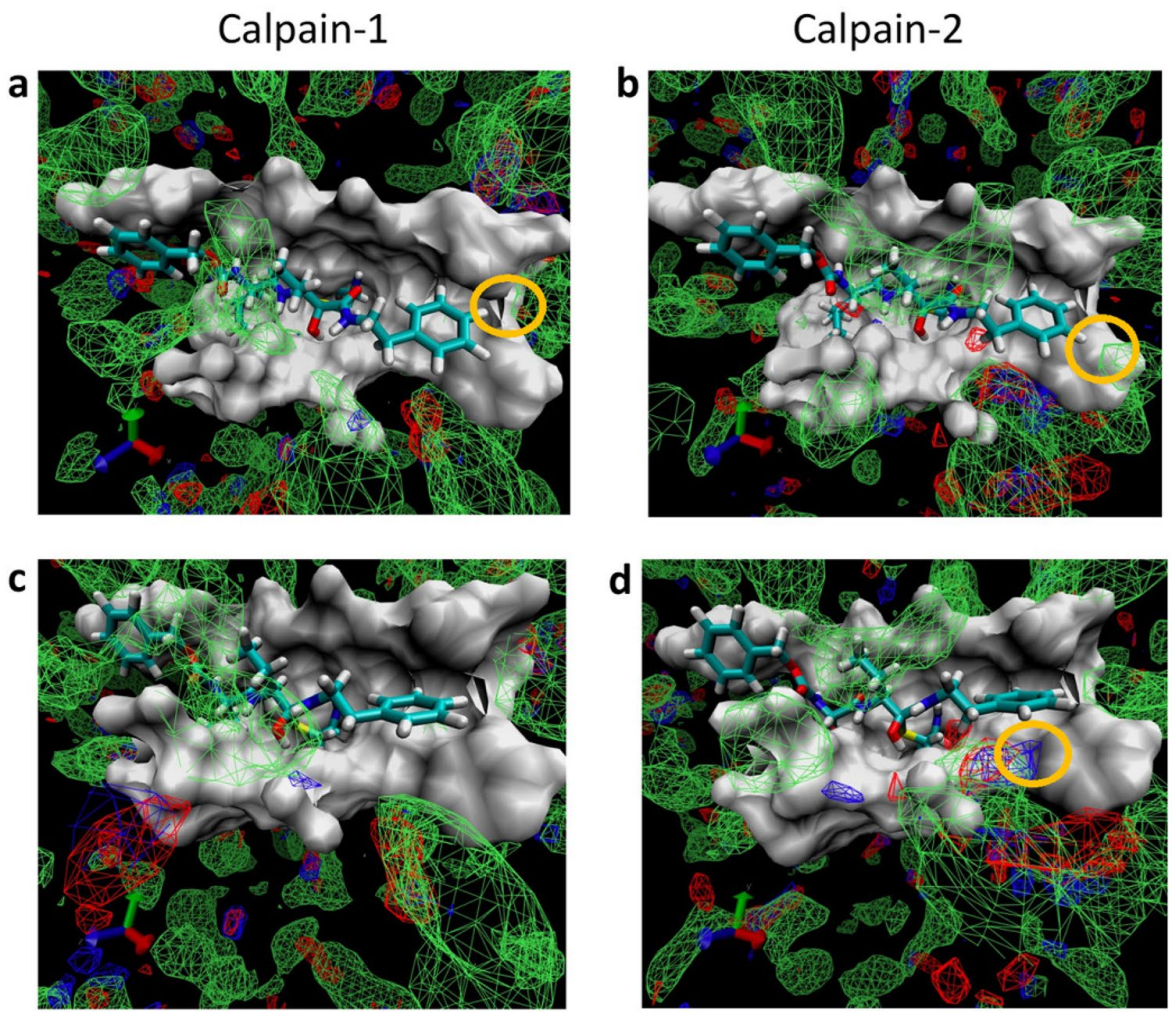
Comparison between SILCS FragMaps of calpain-1 (**a**, **c**) and calpain-2 (**b**, **d**). Green mesh density is polar FragMap, blue is hydrogen bond donor FragMap, and red is hydrogen bond acceptor FragMap. The cutoff value for all density mesh is set to be − 1. Yellow circles indicate that additional polar group is favored at para-position in calpain-1 P1’ site (**a**), but at meta-position in calpain-2 P1’ site (**b**); no density was found at ortho-position in calpain-1 P1’ site (**c**), but hydrogen bond donor and acceptor densities were present at ortho-position in calpain-2 (**d**, yellow circled area)

To facilitate the structure-based design, we conducted the Site Identification by Ligand Competitive Saturation (SILCS) simulations [55] to generate fragment density maps on calpain-1 and calpain-2 protein surface. During SILCS simulations, each protein was soaked in aqueous solution with a library of small functional groups. By allowing competitive and reversible binding of these groups to the flexible protein surface, SILCS generates a three-dimensional probability map for molecular fragments (FragMap). As shown in Fig. 4, at P1’ site, additional substitution is favored at para-position of phenyl group in calpain-1 P1’ site, but at meta-position in calpain-2 P1’ site; hydrogen bond donor and acceptor were present at ortho-position in calpain-2, but no density was found at ortho-position in calpain-1 P1’ site.

**Fig. 5:**
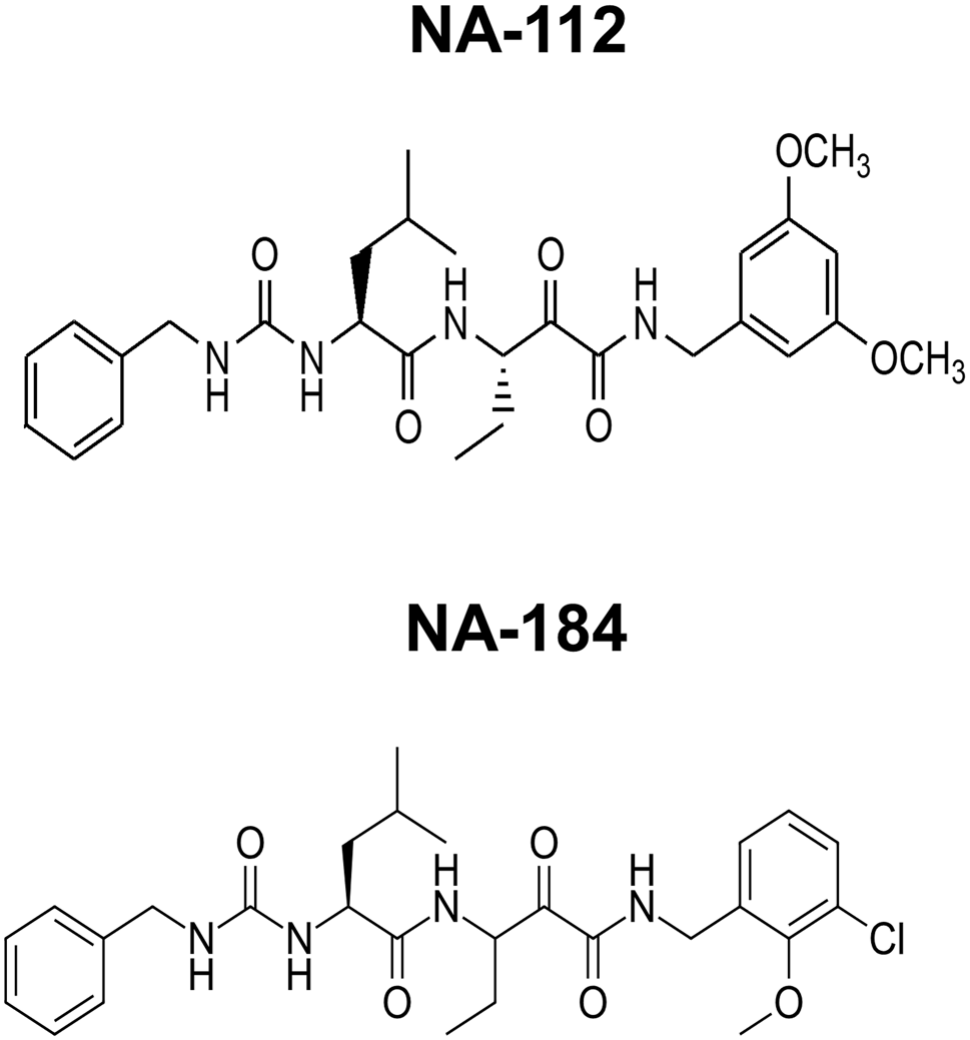
Structure of the selective calpain-2 inhibitors NA-112 and NA-184

Based on these findings, we synthesized about 130 molecules, which were first tested for their affinity/selectivity against human calpain-1 and calpain-2. Among these 130 molecules, two of them looked very promising, NA-112 and NA-184, since they have a low Ki against calpain-2 and exhibit the best selectivity between calpain-1 and calpain-2 (Fig. 5). The increased calpain-2 selectivity of NA-184 agrees well with the prediction of SILCS FragMaps in Fig. 4. Substitutions on P3 site aromatic ring did not increase selectivity, likely due to the high mobility of the P3 site. Replacing carbamate linker by urea linker on P3 site increased the overall binding pose stability and also metabolic stability. We verified that these 2 inhibitors cross the BBB, as assessed by the inhibition of endogenous calpain-2 and found that they have more than 20-fold selectivity against calpain-2 than against calpain-1 in vivo. Both molecules provide significant neuroprotection in the CCI mouse model of TBI and inhibit the increase in blood levels of P13BP 24 h after TBI (Baudry et al., in preparation).

We have further studied the pharmacokinetics and pharmacodynamics of NA-184. In mice, NA-184 has a half-life of 5.15 h in plasma following i.v. administration and a half-life of 3.5 h in brain. However, its half-life for calpain-2 inhibition in brain is about 7.5 h, which is likely due to the fact that NA-184 forms a reversible covalent bond with calpain-2, which is slowly dissociating [56]. We are therefore proposing to develop NA-184 as a potential therapeutic treatment for TBI.

NA-184 has an in vivo IC_50_ of 130 nM against mouse calpain-2 and exhibits an ED50 of 0.13 mg/kg against cell death in the CCI mouse model of TBI, with no inhibition of calpain-1 at doses up to 10 mg/kg. When tested against human calpain-2 and spectrin degradation, NA-184 IC_50_ is 1.3 nM and no inhibition of human calpain-1 is detected at concentrations up to 10 µM. Thus, NA-184 is very potent and selective human calpain-2 inhibitor. We have established a validated synthesis route for NA-184, which has been used to generate the batches of NA-184 required for the pre-IND studies. We have also found that NA-184 is effective at inhibition of brain calpain-2 activity following i.p., i.v., intranasal, and subcutaneous injection, but efficacy is more limited when administered by oral gavage (Baudry et al., in preparation).

## Conclusions and Clinical Plans

As indicated above, we have selected NA-184 as our clinical candidate to be further developed for the treatment of TBI. We are currently performing the pre-IND studies, including safety and toxicity, with Altasciences. When these are completed, we will submit an IND application to the FDA in order to be authorized to initiate a phase Ia/Ib clinical trial. The clinical interventions planned are (1) a phase Ia clinical trial in healthy volunteers that will consist in a single ascending dose (SAD) injection to assess the safety, tolerability, and pharmacokinetics of NA-184 and (2) a phase Ib clinical trial that will consist in a multiple ascending dose (MAD) study evaluating the effects of NA-184 in patients with acute TBI. The phase Ib study will be a double-blind, placebo-controlled, multiple ascending study to evaluate two dose levels in hospitalized patients with acute TBI. Dose levels will be selected based on the single ascending dose study findings. Patients admitted to the trauma center who meet entrance criteria will receive their first dose of study drug within 24 h of documented TBI. Patients will receive twice daily dosing for 5 consecutive days. Study medication will be administered as an i.v. infusion over 20 min followed by collection of safety, tolerability, and pharmacokinetic data.

The primary outcome measures of the phase Ia will be the incidence of adverse effects and the secondary outcomes will be the pharmacokinetics of NA-184 in serum. For the phase Ib, the primary outcomes will also be the incidence of adverse effects and the plasma levels of Nfl, as an indicator of neuronal damage. Secondary outcomes will also be the pharmacokinetics of NA-184 in serum, and other outcome measures will be the comparison of various blood-based biomarkers at days 3, 5, and 7 days after TBI (P13BP, SNTF, GFAP). We anticipate that these studies will be completed by end of 2025.

This trial will be the first clinical trial for a selective calpain-2 inhibitor for the treatment of TBI. Since we have obtained other preclinical data indicating that such inhibitors could also be very beneficial to prevent the long-term pathological consequences of seizure activity [57], positive results would open avenues for expanding the clinical applications of selective calpain-2 inhibitors.
